# A randomized controlled trial on the effects of antenatal exercise on birth weight and neonatal body composition

**DOI:** 10.1186/1687-9856-2015-S1-O38

**Published:** 2015-04-28

**Authors:** Sumudu Seneviratne, Graham Parry, Yannan Jiang, Lesley Mc Cowan, Wayne Cutfield, Raquel Rodrigues, Silmara Gusso, Alec Ekeroma, Susan Craigie, Paul Hofman

**Affiliations:** 1 Liggins Institute, University of Auckland, Auckalnd, New Zealand; 2 Gravida, Auckland, New Zealand; 3 Department of Obstetrics and Gynaecology, University of Auckland, Auckland, New Zealand

## Aims

To determine the effects of an antenatal exercise programme on birth weight and neonatal body composition of offspring of overweight and obese women.

## Methods

We are conducting a parallel arm randomized controlled clinical trial in Auckland, New Zealand (NZ). Eligible participants were enrolled to the study from March 2013 to April 2014. The intervention group participated in a 16-week home-based moderate-intensity exercise programme utilising stationary cycles and heart rate monitors. Maternal measures including weight, aerobic fitness, physical activity and diet were assessed at baseline and end of intervention. Neonatal and maternal body composition were assessed 14 days after delivery.

## Results

A total of 75 participants were recruited (intervention n =37; control n = 38). Participants had a mean pre-pregnancy BMI of 31.5 kg/m^2:^; age of 30.5 years and weight of 91 kg at study entry. 57% were NZ European/other, 29% Pacific Island and 13% Maori. 25% were nulliparous. Maternal characteristics known to affect birth weight were similar between groups at baseline. To date, 42 participants (intervention n= 19, control n=23) have completed the study. The birth weight (g) of offspring in the intervention group is 3701 ± 561 compared to 3552 ± 495 in the control group. Secondary offspring outcomes in the exercise and control groups are: birth length (cm) 51.5 vs 51.6; head circumference(cm) 35.1 vs 34.8; customised birth weight centiles 59% vs 42%; neonatal lean mass(g) 3717 vs 3546; neonatal fat mass(g) 367 vs 275 and neonatal %body fat 8.8% vs 7.2%. Maternal outcomes in exercise (n=24) and control (n= 26) groups are: weight gain (kg) over the intervention period 8.93 vs 8.94 ; postpartum maternal BMI 32.7 vs 35.5 and % body fat 46% vs 48%.

## Conclusion

This is the first clinical trial exploring the effects of antenatal exercise in overweight and obese women on neonatal body composition Preliminary trial results on 42 participants indicate a trend towards increased neonatal fat mass and % body fat in the intervention group. Trial data collection will be completed by October 2014.

